# Enhanced PM_2.5_ pollution in China due to aerosol-cloud interactions

**DOI:** 10.1038/s41598-017-04096-8

**Published:** 2017-06-30

**Authors:** Bin Zhao, Kuo-Nan Liou, Yu Gu, Qinbin Li, Jonathan H. Jiang, Hui Su, Cenlin He, Hsien-Liang R. Tseng, Shuxiao Wang, Run Liu, Ling Qi, Wei-Liang Lee, Jiming Hao

**Affiliations:** 1 Joint Institute for Regional Earth System Science and Engineering and Department of Atmospheric and Oceanic Sciences, University of California, Los Angeles, CA 90095 USA; 2 Jet propulsion Laboratory, California Institute of Technology, Pasadena, California 91109 USA; 3 State Key Joint Laboratory of Environment Simulation and Pollution Control, School of Environment, Tsinghua University, Beijing, 100084 China; 4 State Environmental Protection Key Laboratory of Sources and Control of Air Pollution Complex, Beijing, 100084 China; 5 Research Center for Environmental Changes, Academia Sinica, Taipei, Taiwan

## Abstract

Aerosol-cloud interactions (aerosol indirect effects) play an important role in regional meteorological variations, which could further induce feedback on regional air quality. While the impact of aerosol-cloud interactions on meteorology and climate has been extensively studied, their feedback on air quality remains unclear. Using a fully coupled meteorology-chemistry model, we find that increased aerosol loading due to anthropogenic activities in China substantially increases column cloud droplet number concentration and liquid water path (LWP), which further leads to a reduction in the downward shortwave radiation at surface, surface air temperature and planetary boundary layer (PBL) height. The shallower PBL and accelerated cloud chemistry due to larger LWP in turn enhance the concentrations of particulate matter with diameter less than 2.5 μm (PM_2.5_) by up to 33.2 μg m^−3^ (25.1%) and 11.0 μg m^−3^ (12.5%) in January and July, respectively. Such a positive feedback amplifies the changes in PM_2.5_ concentrations, indicating an additional air quality benefit under effective pollution control policies but a penalty for a region with a deterioration in PM_2.5_ pollution. Additionally, we show that the cloud processing of aerosols, including wet scavenging and cloud chemistry, could also have substantial effects on PM_2.5_ concentrations.

## Introduction

The interactions between aerosols and clouds play important roles in both meteorological variation and climate change, which represent the single largest uncertainty in anthropogenic radiative forcing of Earth’s climate^1^. Specifically, aerosols act as cloud condensation nuclei (CCN) or ice nucleating particles (INP), modifying cloud physical and radiative properties and precipitation forming processes^2–4^. Changes in cloud and radiation fields subsequently affect other meteorological variables, such as surface air temperature^5, 6^, diurnal temperature range^7^, planetary boundary layer (PBL)^8^, and wind speed^9, 10^. Such meteorological variations further induce feedback on regional air quality, for example, surface ozone and aerosol concentrations^11^.

Many studies have investigated the effects of aerosol-cloud interactions on meteorology and climate^12–17^. However, only limited studies have elucidated the aerosol-cloud feedback from the air quality perspective^6, 10, 18–21^, which requires running a fully coupled meteorology-chemistry model. Among them, it has been a common practice^10, 18–20^ to assess the aerosol-cloud interaction impact on air quality by comparing the baseline scenario with a hypothetical scenario assuming a prescribed vertically uniform cloud droplet number concentration (CDNC) of 250 cm^−3^, consistent with the treatment in the Weather Research and Forecasting (WRF) model without coupling with chemistry. While some other studies^6, 22^ have used different prescribed CDNC or CCN distributions, these hypothetical scenarios generally represent a rather polluted condition with a substantial amount of aerosols^23–25^. In this case, the physical meaning of simulation results (i.e., the difference between the baseline and hypothetical scenarios) does not actually represent the effects of aerosol-cloud interactions (or aerosol indirect effect). In the context of climate study, the impact of aerosol-cloud interactions usually means the impact of increased aerosols (compared to a pristine condition or the preindustrial era) due to human activities by interacting with clouds. To the best of our knowledge, none of the previous studies have evaluated the impact of increased anthropogenic aerosols on air quality through aerosol-cloud interactions. A systematic assessment of this impact will enhance our understanding of this “indirect pathway” through which human activities affect PM_2.5_ pollution in addition to the “direct pathway” involving primary emissions and chemical formation of PM_2.5_.

In this study, we investigate the impact of anthropogenic aerosols on meteorology and air quality through aerosol-cloud interactions in China in January and July 2013, using the Weather Research and Forecasting Model with Chemistry (WRF-Chem), a fully coupled meteorology-chemistry model. We select China as the target region because the aerosol loadings in China have been among the highest in the world^26^, with daily PM_2.5_ (particulate matter with diameter less than 2.5 μm) concentrations exceeding 500 μg m^−3^ from time to time^27^. The high aerosol loadings could have strong and unparalleled influence on regional climate and air quality through aerosol-cloud-radiation interactions^10, 28, 29^. We find that increased aerosols could lead to an additional enhancement in PM_2.5_ concentrations through a positive feedback loop induced by aerosol-cloud interactions, which is responsible in part for the severe PM_2.5_ pollution in China. The differences between our assessment results and previous studies, and the underlying reasons are elucidated.

## Results

### Model evaluation

The WRF-Chem version 3.7.1 has been applied over a domain covering most of China except for some sparsely populated regions in westernmost and northernmost China (Supplementary Figure 1). The configuration of the modeling system is detailed in the Methods section. Since a reasonable model representation of meteorological and chemical variables would lay the foundation for evaluating the aerosol-cloud interactions, we compare model simulations with a series of surface meteorology, surface air quality, and satellite observational datasets (see the Methods section). Below we summarize some key evaluation results, and more details are provided in Supplementary Section 1. The model predictions agree fairly well with surface meteorological observations. Table 1 shows that the performance statistics for wind speed at 10 m (WS10) in July and water vapor mixing ratios at 2 m (Q2) in January and July are within or very close to the benchmark ranges proposed by Emery *et al*.^30^. Note that these benchmark values are proposed based on the performance of a series of model simulations with four dimensional data assimilation (FDDA). Nevertheless, FDDA is not utilized here to allow full aerosol-cloud interactions, therefore the model performance is not expected to be as good as those with FDDA. The WS10 in January and temperature at 2 m (T2) in both months exceed the benchmark range but still have smaller or similar biases compared with most previous WRF-Chem applications without FDDA over East Asia^10, 31–35^.

**Table 1:** Statistics of model performance for meteorological and chemical predictions for the baseline scenario (BASE).

Variable	Observations	Month	Mean Obs	Mean Sim	MB	GE	RMSE	IOA
WS10 (m s^−1^)	NCDC	January	2.54	3.33	0.79	1.68	2.27	0.66
		July	2.70	3.12	0.42	1.52	2.02	0.64
T2 (K)		January	277.4	276.1	−1.28	3.66	4.60	0.94
		July	298.4	297.2	−1.17	2.80	3.63	0.91
Q2 (g kg^−1^)		January	3.94	3.07	−0.87	1.52	2.54	0.81
		July	15.93	14.87	−1.06	2.17	3.14	0.90
Precipitation (mm month^−1^)	GPCC	January	10.2	12.6	2.3	9.5	20.9	0.62
		July	170.4	182.0	11.6	92.8	147.5	0.79
			**Mean Obs**	**Mean Sim**	**NMB**	**NME**	**MFB**	**MFE**
PM_2.5_ (μg m^−3^)	MEP	January	129.0	115.4	−11%	36%	−19%^b^	40%^b^
		July	39.2	39.5	1%	33%	−7%^b^	39%^b^
SO_2_ (μg m^−3^)		January	81.8	74.2	−9%	63%	−10%	59%
		July	20.0	29.7	49%	93%	12%	65%
NO_2_ (μg m^−3^)		January	62.8	53.0	−16%	28%	−22%	34%
		July	28.4	33.8	19%	53%	2%	48%
Daily max O_3_ (μg m^−3^)		January	86.7	68.7	−21%	31%	−21%	34%
		July	127.9	126.6	−1%	22%	−1%	24%
			**Mean Obs**	**Mean Sim**	**NMB**	**NME**	**RMSE**	**R**
SWD (W m^−2^)	CERES	January	118.2	139.2	18%	19%	31.1	0.84
		July	219.9	244.6	11%	19%	49.4	0.77
LWD (W m^−2^)		January	238.4	218.5	−8%	9%	24.3	0.98
		July	381.5	368.4	−3%	4%	19.2	0.96
NO_2_ column (10^15^ molc cm^−2^)	OMI	January	5.43	5.31	−2%	40%	4.35	0.90
		July	1.69	1.82	8%	51%	1.83	0.74
AOD	MODIS/TERRA	January	0.48	0.40	−16%	38%	0.27	0.56
		July	0.36	0.31	−15%	47%	0.23	0.66
LWP (g m^−2^)		January	132.2	54.1	−59%	63%	97.1	0.67
		July	181.1	48.0	−74%	76%	150.2	0.40
CDNC (cm^−3^)		January	73.5	51.9	−29%	67%	72.2	0.62
		July	30.3	18.8	−38%	65%	25.0	0.51
CF		January	0.63	0.43	−32%	38%	0.29	0.55
		July	0.69	0.63	−9%	25%	0.21	0.65

^a^WS10, wind speed at 10 m; T2, temperature at 2 m; Q2, water vapor mixing ratios at 2 m; SWD, downward shortwave radiation at surface; LWD, downward longwave radiation at surface; AOD, aerosol optical depth; LWP, liquid water path; CDNC, cloud droplet number concentration; CF, cloud fraction; MB, mean bias; GE, gross error; RMSE, root mean square error; IOA, index of agreement; NMB, normalized mean bias; NME, normalized mean error; R, correlation coefficient; MFB, mean fractional bias; MFE, mean fractional error; NCDC, National Climatic Data Center; CERES, Clouds and the Earth’s Radiant Energy System; GPCC, Global Precipitation Climatology Center; OMI, Ozone Monitoring Instrument; MODIS, Moderate Resolution Imaging Spectroradiometer; MEP, Ministry of Environmental Protection of China.

^b^Boylan and Russell^36^ proposed a model performance criteria of MFE ≤ +75% and MFB ≤ ±60%, and a model performance goal of MFE ≤ +50% and MFB ≤ ±30%.

**Figure 1:**
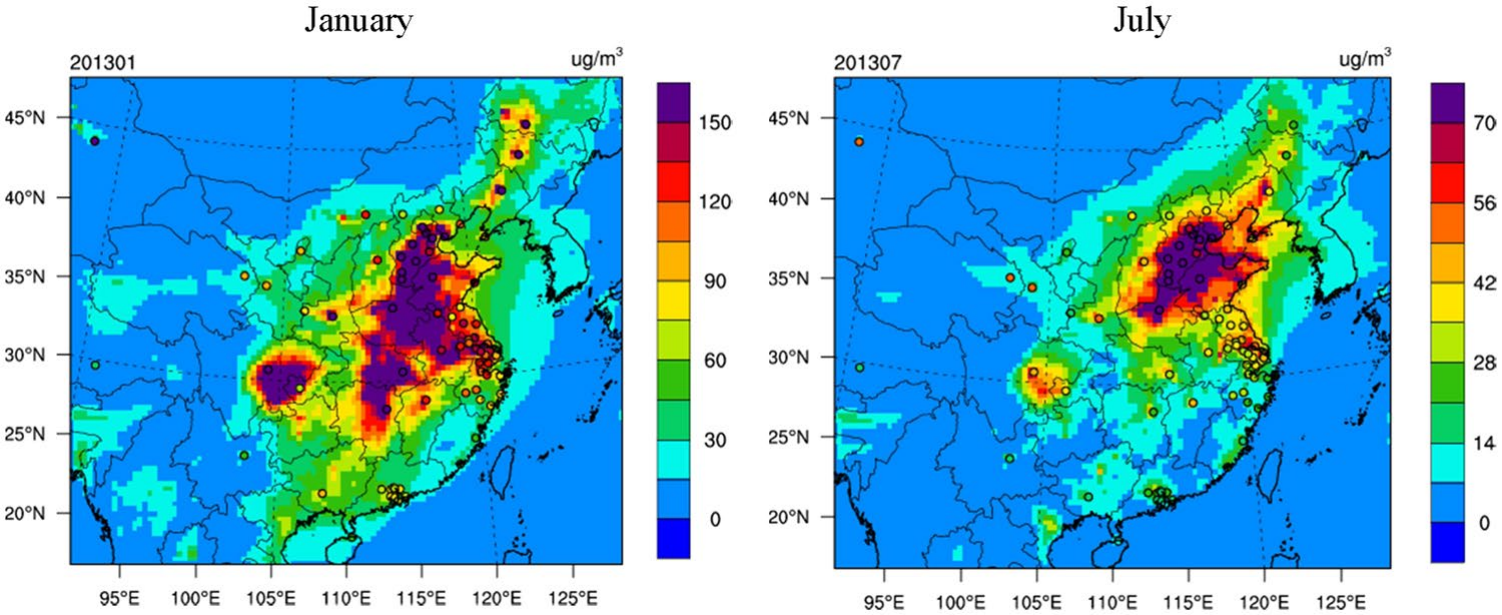
Observed (dots) and simulated (contours) monthly mean PM_2.5_ concentrations in the BASE scenario in January (left panel) and July (right panel), 2013. This figure is produced using the NCAR Command Language (Version 6.2.1) [Software]. (2014). Boulder, Colorado: UCAR/NCAR/CISL/TDD. http://dx.doi.org/10.5065/D6WD3XH5.

With regard to surface air quality, Table 1 shows that the model well captures the average PM_2.5_ concentrations in 74 major cities of China in both months, with NMBs of −11% and 1% in January and July, respectively. The performance statistics for PM_2.5_ meet the model performance goal (MFB within ±30% and MFE ≤ 50%) proposed by Boylan and Russell^36^ in both months. Figure 1 further overlays the observed and simulated monthly average PM_2.5_ concentrations in 74 major cities. The model well reproduces the spatial pattern of PM_2.5_ concentrations, particularly high concentration levels over the North China Plain, the Sichuan Basin, the Yangtze River Delta, and Hubei-Hunan provinces. The PM_2.5_ concentrations are underestimated in relatively remote regions, including the western China and the northeastern China, though this study focuses on the more contaminated Eastern and Central China (ECC, see Supplementary Figure 1). Moreover, PM_2.5_ concentrations are moderately overestimated in the Sichuan Basin in both months and in the Pearl River Delta in July. We also compare simulated concentrations of gaseous pollutants and PM_2.5_ chemical components with observations, and find reasonably good model performance (see Supplementary Section 1).

We also evaluate model simulations against satellite observations (Table 1; Supplementary Figure 2). The model captures the spatial pattern and magnitude of NO_2_ vertical column density quite well except for the overestimates in southern China in July. The aerosol optical depth (AOD) is also reproduced fairly well, with slight (~15%) underestimates in both months, probably due to underestimates in dust emissions and secondary organic aerosol (SOA) formation. The simulation results of cloud properties are subject to larger biases compared with other variables. The cloud fraction (CF) is underestimated by 32% and 9% in January and July, respectively, while the liquid water path (LWP) is substantially underestimated by over 50%, which is a common problem for many chemical transport simulations^31, 33, 37, 38^. It is noted, however, the LWP retrieved from Moderate Resolution Imaging Spectroradiometer (MODIS) may be biased by a factor of 2 due to uncertainties in cloud particle size assumption^39^. The discrepancies in cloud parameters may be related to several factors including aerosol number concentrations, water vapor, aerosol activation parameterization, cloud microphysics, and cumulus cloud schemes (see Supplementary Section 1).

### Impact of aerosol-cloud interactions on meteorology and air quality

The approach to assess the effects of aerosol-cloud interactions is to design a hypothetical scenario without aerosol-cloud interactions and compare with the baseline scenario where these interactions are accounted for. The most common approach^6, 10, 18–20, 22^ to design the hypothetical scenario has been to assume a prescribed distribution of CCN or CDNC, with the latter being more frequently used. Since the objective of this study is to assess the aerosol-cloud interaction impact associated with anthropogenic aerosols, it is necessary to contrast the baseline WRF-Chem simulation with a hypothetical scenario representing a pristine environment. For this reason, we design a scenario named “PRSC10” (see Table 2) by assuming a vertically uniform CDNC of 10 cm^−3^, which is roughly consistent with liquid clouds in clean ocean conditions, according to satellite observations and global simulations^23–25^. Note that a number of studies^14, 40, 41^ have created a pristine condition in the hypothetical scenario by prescribing a low AOD or aerosol number concentration, or by eliminating the anthropogenic particulate matter (PM) emissions and secondary PM formations^9, 37, 38^; however, these types of numerical experiments cannot be utilized to assess the feedback of aerosol-cloud interactions on PM_2.5_ concentrations. Besides “PRSC10”, we design two scenarios for sensitivity analysis. As described in the introduction section, previous studies^10, 18–20^ mostly evaluated the aerosol-cloud interaction impact by comparing the baseline scenario with a hypothetical scenario assuming a prescribed vertically uniform CDNC of 250 cm^−3^, which represents a rather polluted condition. We have also designed such a hypothetical scenario (“PRSC250”, see Table 2) in order to compare our assessment results with those determined from the numerical experiment used in previous studies. Moreover, an additional hypothetical scenario (“PRSC10_NWDAQ”, see Table 2) has also been designed to examine the effects of cloud processing of aerosols, including wet scavenging and cloud chemistry. The PRSC10_NWDAQ scenario is the same as the PRSC10 scenario except that the cloud chemistry and wet scavenging schemes are turned off.

**Table 2:** Scenarios for evaluation of the impact of aerosol-cloud interactions.

Scenario name	Scenario definition	Note
BASE	The default WRF-Chem v3.7.1	
PRSC10	The same as the BASE scenario except that a prescribed vertically uniform CDNC of 10 cm^−3^ is used	The difference between the BASE and PRSC10 scenarios represents the impact of aerosol-cloud interactions due to anthropogenic aerosols
PRSC250	The same as the BASE scenario except that a prescribed vertically uniform CDNC of 250 cm^−3^ is used	The difference between the BASE and PRSC250 scenarios represents the impact of aerosol-cloud interactions compared to a rather polluted condition with uniform CDNCs, which is consistent with the treatment in WRF without coupling with chemistry
PRSC10_NWDAQ	The same as the PRSC10 scenario except that wet scavenging and cloud chemistry are deactivated	The difference between the BASE and PRSC10_NWDAQ scenarios represents overall impact of aerosol-cloud interactions due to anthropogenic aerosols, and the cloud processing of aerosols, i.e., wet scavenging and cloud chemistry

**Figure 2:**
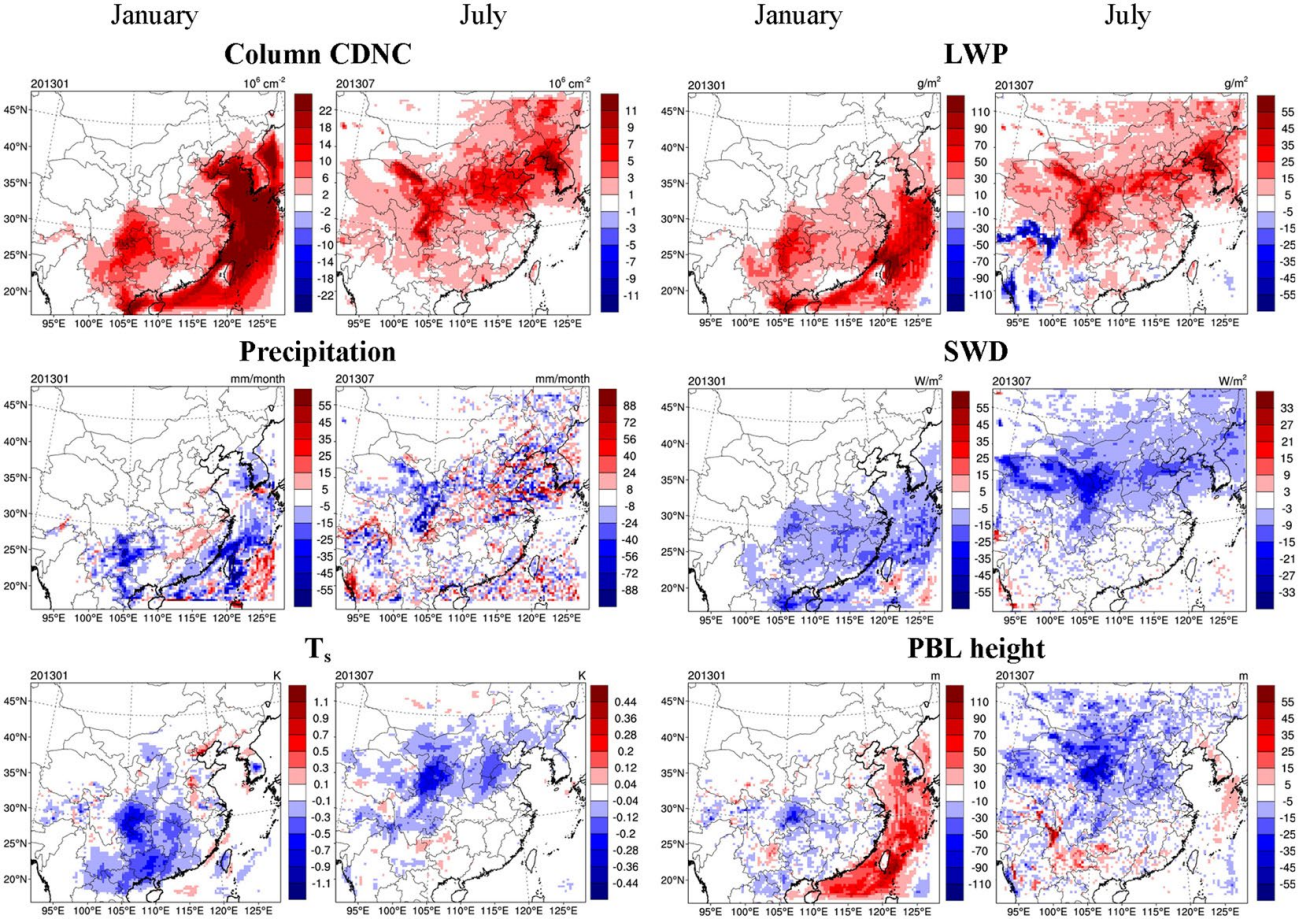
Impact of anthropogenic aerosols on meteorological variables through aerosol-cloud interactions, determined from the scenarios of BASE and PRSC10 (BASE minus PRSC10). The meteorological variables considered are column cloud droplet number concentration (CDNC), liquid water path (LWP), precipitation, downward shortwave radiation at surface (SWD), surface air temperature (T_s_), and planetary boundary layer (PBL) height. This figure is produced using the NCAR Command Language (Version 6.2.1) [Software]. (2014). Boulder, Colorado: UCAR/NCAR/CISL/TDD. http://dx.doi.org/10.5065/D6WD3XH5.

Figure 2 illustrates the impact of aerosol-cloud interactions due to anthropogenic aerosols on meteorological variables, determined from the differences between the BASE and PRSC10 scenarios. The aerosol-cloud interaction impact is initiated by the difference in CDNCs. Figure 2 shows that the prognostic column CDNC (i.e., vertically integrated CDNC) in the BASE scenario is significantly larger than the prescribed values in the PRSC10 scenario. Larger CDNC is associated with smaller droplet size (“first indirect effect”)^2^, which could delay precipitation formation and thus increase cloud water (“second indirect effect”)^3, 4^. As a result, LWP increases in most of the domain in both months when aerosol-cloud interactions are included in the model. An exception is that LWP decreases in July along the Himalaya Mountain where the aerosol concentration in the upper air is so low that the prognostic upper-air CDNC in the BASE scenario is even smaller than the prescribed value of 10 cm^−3^ used in the PRSC10 scenario, leading to a larger cloud droplet size and smaller LWP in the BASE scenario. The spatial pattern of LWP changes is strongly dependent on the distribution of cloud water amount. In January, high LWP, and hence large LWP changes, occur in southern China and over the ocean, while large LWP changes occur in northern and western China in July (Fig. 2 and Supplementary Figure 2). On average, LWP increases by 18.4 g m^−2^ and 10.9 g m^−2^ over the ECC region (defined in Supplementary Figure 1) in January and July, respectively. Precipitation is suppressed in the majority of the domain because of a delayed onset of precipitation as described above. It is noted that precipitation can also be enhanced in some regions due to the horizontal transport and mass balance of water vapor. The aerosol-induced cloud changes further lead to a reduction in downward shortwave radiation at surface (SWD) in most of the domain because of the enhanced scattering and absorption of incoming solar radiation by clouds. Factors contributing to the SWD reduction include (1) the increase in LWP, and (2) the existence of more and smaller cloud droplets for a constant LWP. The spatial pattern of SWD reductions corresponds well with that of LWP increases, confirming the causality described above. The reduction in SWD suppresses surface energy fluxes and decreases surface air temperature (T_s_), which further inhibits vertical mixing and leads to a shallower PBL on land (Fig. 2).

**Figure 3:**
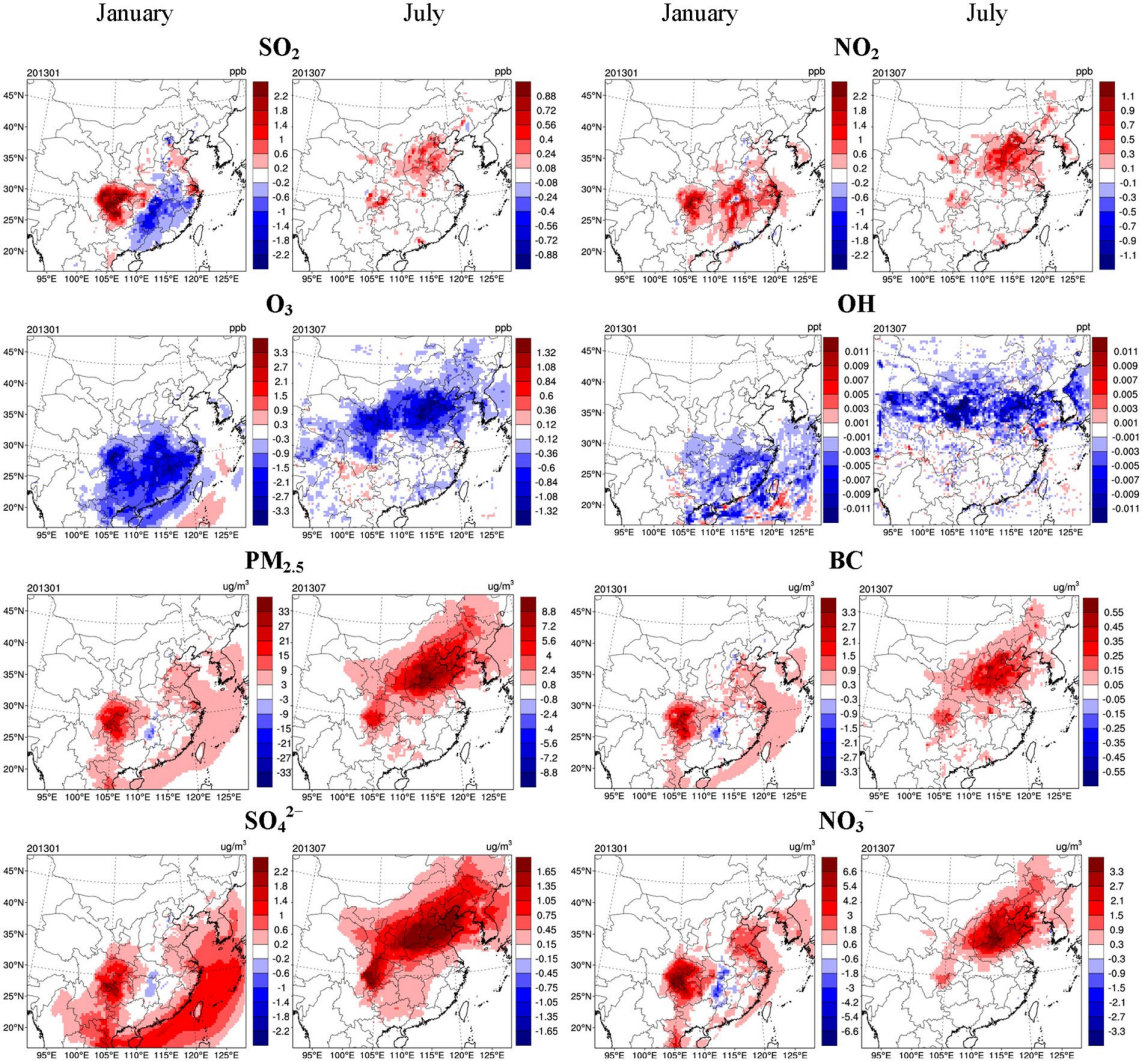
Same as Fig. 2, but for concentrations of gaseous pollutants, PM_2.5_, and major PM_2.5_ chemical components, including black carbon (BC), SO_4_^2–^, and NO_3_^−^. This figure is produced using the NCAR Command Language (Version 6.2.1) [Software]. (2014). Boulder, Colorado: UCAR/NCAR/CISL/TDD. http://dx.doi.org/10.5065/D6WD3XH5.

The aerosol-induced meteorological changes in turn exert feedback on the changes in air quality. Figure 3 illustrates the impact of aerosol-cloud interactions on concentrations of gaseous pollutants and PM_2.5_ along with major chemical constituents, based on the BASE and PRSC10 scenarios. The inclusion of aerosol-cloud interactions enhances SO_2_ and NO_2_ concentrations in most of the domain due largely to a shallower PBL. Meanwhile, SO_2_ concentrations are found to decrease in some regions in January probably due to enhanced cloud chemistry in conjunction with increased LWP. The inclusion of aerosol-cloud interactions results in significant decrease in O_3_ concentrations in the vast majority of the domain due largely to weakened SWD and thus reduced photolysis rate.

As a result of aerosol-cloud interactions, PM_2.5_ concentrations increase remarkably in the majority of the domain. The increases average to 3.7 μg m^−3^ (5.3%) and 2.2 μg m^−3^ (10.3%) over the ECC region, with maximums of 33.2 μg m^−3^ (25.1%) and 11.0 μg m^−3^ (12.5%) in January and July, respectively. In addition to total PM_2.5_, the concentrations of major PM_2.5_ chemical components, including BC, SO_4_^2–^, and NO_3_^−^, also show pronounced increases in most of the domain. The changes in various components present fairly similar spatial patterns, indicative of the dominant role of shallower PBL in enhancing PM_2.5_ concentrations. The spatial pattern of PM_2.5_ changes, however, is considerably different from that of PBL changes. This is because the magnitude of PM_2.5_ changes is significantly affected by not only PBL changes but also the absolute PM_2.5_ concentrations. As described above, the spatial pattern of PBL changes is closely tied to the distribution of cloud water amount, while the distribution of PM_2.5_ concentrations is mainly determined by emission intensity, which is usually not correlated with cloud water amount. For this reason, large PM_2.5_ changes occur in regions that overlap with large PM_2.5_ concentrations and cloud water amount. In other words, a large change in meteorological parameters, including LWP, SWD and PBL height (e.g., over the ocean in January and over Northeast Asia in July), does not necessarily lead to a large change in PM_2.5_ concentrations. Also, the urban-to-rural gradient of PM_2.5_ changes is considerably smaller than that of the absolute PM_2.5_ concentrations, since the changes in PBL exhibit little difference between urban and rural areas.

**Figure 4:**
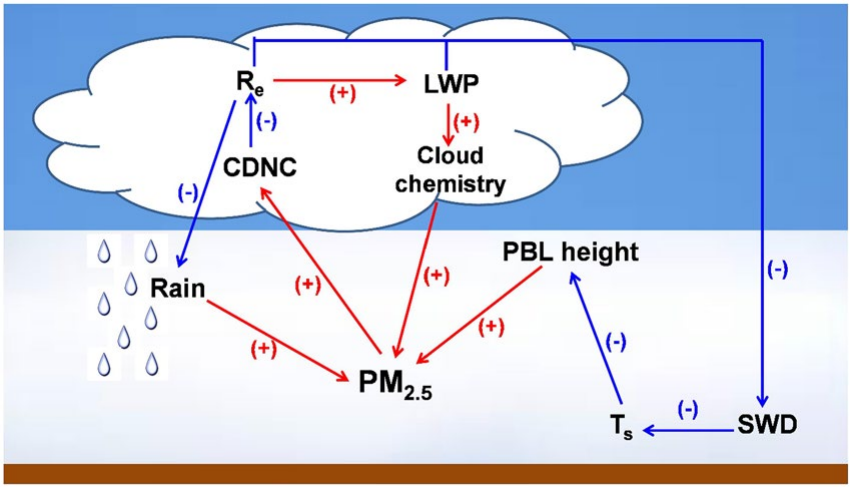
Schematic diagram for the “self-enhancement” of PM_2.5_ due to aerosol-cloud interactions. The (+)/(−) in the figure means an increase in PM_2.5_ would lead to an increase/decrease in a certain variable. R_e_ represents cloud droplet effective radius.

While changes in PBL plays a predominant role in PM_2.5_ enhancement, accelerated cloud chemistry due to larger LWP also makes a noticeable contribution, as indicated by the significant increase in SO_4_^2–^ concentrations over the ocean in January. Moreover, the suppression in precipitation also favors the accumulation in PM_2.5_ concentrations. Based on the analysis above, it is apparent that increased aerosols due to anthropogenic activities alter cloud properties and thus result in changes in PBL height, precipitation, and cloud chemistry, which in turn enhance PM_2.5_ concentrations. This “self-enhancement”, as illustrated using a schematic diagram in Fig. 4, amplifies the magnitude of initial perturbations in PM_2.5_ concentrations.

**Figure 5:**
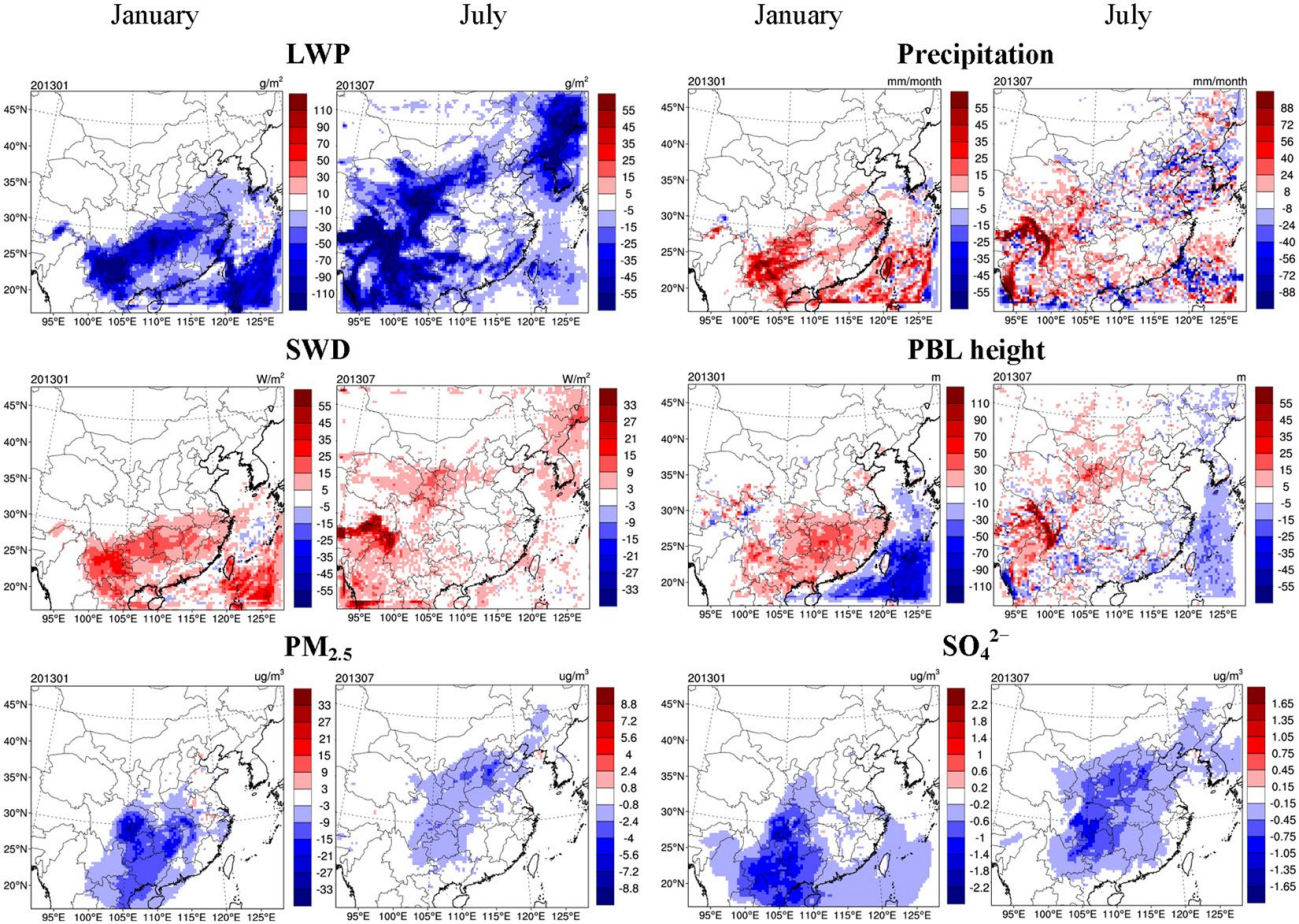
Impact of aerosol-cloud interactions on meteorological variables (LWP, precipitation, SWD, and PBL height) and concentrations of PM_2.5_ and SO_4_^2–^, relative to a polluted condition with a uniform CDNC of 250 cm^−3^. The results are determined from the scenarios of BASE and PRSC250 (BASE minus PRSC250). This figure is produced using the NCAR Command Language (Version 6.2.1) [Software]. (2014). Boulder, Colorado: UCAR/NCAR/CISL/TDD. http://dx.doi.org/10.5065/D6WD3XH5.

Our finding about the PM_2.5_ enhancement due to aerosol-cloud interactions, however, are not consistent with the simulation results in many previous studies^10, 18–21^, which reported that the inclusion of aerosol-cloud interactions mostly resulted in a decline in PM_2.5_ concentrations. In accordance with these studies, we design the PRSC250 scenario (see Table 2) which assumes a prescribed vertically uniform CDNC of 250 cm^−3^, and assess the aerosol-cloud interaction impact using the difference between the BASE and PRSC250 scenarios, as shown in Fig. 5. In contrast to the preceding results (Figs 2 and 3), the LWP changes due to aerosol-cloud interactions turn out to be negative in most of the domain when evaluated by the differences between BASE and PRSC250 scenarios. This is because the PRSC250 scenario is usually more polluted than the BASE scenario in the upper air. While the prognostic CDNCs in the BASE scenario could be larger than the prescribed value of 250 cm^−3^ at lower altitude, it is generally smaller at high altitude where more cloud water resides, due to a rapid decrease of aerosol number concentrations with height. Following the positive feedback illustrated in Fig. 4, the decline in LWP is associated with increase in precipitation, SWD, T_s_, and PBL on land. Consequently, the PM_2.5_ changes due to aerosol-cloud interactions are generally negative when evaluated by the BASE and PRSC250 scenarios, in agreement with the results shown in most previous studies^10, 18–21^. The controlling factor for PM_2.5_ variations is the change in PBL height, followed by changes in cloud chemistry tied to LWP, and in precipitation.

## Discussion

**Figure 6:**
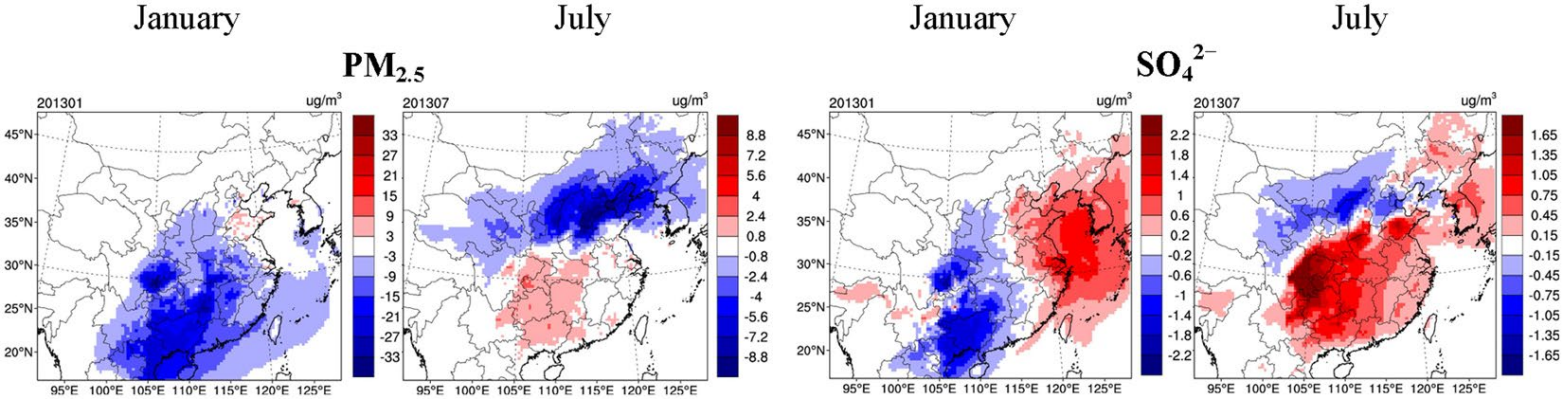
Overall impact of aerosol-cloud interactions due to anthropogenic aerosols, and the cloud processing of aerosols (wet scavenging, cloud chemistry) on concentrations of PM_2.5_ and SO_4_^2–^, determined from the scenarios of BASE and PRSC10_NWDAQ (BASE minus PRSC10_NWDAQ). This figure is produced using the NCAR Command Language (Version 6.2.1) [Software]. (2014). Boulder, Colorado: UCAR/NCAR/CISL/TDD. http://dx.doi.org/10.5065/D6WD3XH5.

The conclusion of this study should be interpreted with care with respect to the definition of “aerosol-cloud interactions”. Here “aerosol-cloud interactions” literally have the same meaning as “aerosol indirect effect”, involving the processes in which aerosols serve as CCN and hence alter cloud micro- and macro-physical properties. The effects of cloud processing of aerosols, including wet scavenging and cloud chemistry, have not been accounted for. To evaluate the potential impact of these processes, we design the PRSC10_NWDAQ scenario (see Table 2) where cloud chemistry and wet scavenging schemes are turned off as well as using a fixed CDNC of 10 cm^−3^. The difference between the BASE and PRSC10_NWDAQ scenarios (shown in Fig. 6) therefore represents the overall impact of the aerosol-cloud interactions (identical to aerosol indirect effects in the present study) and cloud processing of aerosols. Figure 6 reveals that the overall changes in PM_2.5_ turn out to be significantly negative in January. Wet scavenging is known to substantially reduce PM_2.5_ concentrations, whereas cloud chemistry usually elevates PM_2.5_ concentrations (mainly SO_4_^2–^ concentrations). Therefore, the decrease in PM_2.5_ concentrations is mainly attributed to the role of wet scavenging. The corresponding PM_2.5_ changes in July are significantly negative over northern China, but are positive in most of southern China, indicative of a dominant role of wet scavenging and cloud chemistry over northern and southern China, respectively. The predominant impact of cloud chemistry in southern China in July is further confirmed by the substantial increase in SO_4_^2−^ concentrations (Fig. 6). In summary, the wet scavenging and cloud chemistry could have substantial effects on PM_2.5_ concentrations, which vary according to seasons and regions.

The scientific findings of this study have important implications for both research and decision making. It has been well understood that the aerosol-radiation interactions result in surface dimming and stabilization of PBL, which further enhances PM_2.5_ concentrations^27, 42, 43^. Our study indicates that increased aerosol loading could lead to an additional PM_2.5_ enhancement by affecting the formation and microphysical properties of clouds. This “self-enhancement” helps to explain the extremely high PM_2.5_ concentrations during heavy pollution periods, which cannot be reproduced by most chemical transport models without fully coupled meteorology and chemistry^27, 44^. In addition, the “self-enhancement” of initial PM_2.5_ perturbation could translate into an additional air quality benefit under effective pollution control policies, but a penalty for a region experiencing deterioration in PM_2.5_ pollution.

The present study still has a number of limitations. First, the current WRF-Chem does not incorporate SOA formation, explicit aerosol-cumulus cloud interactions, and explicit aerosol effects on ice nucleation, which could introduce uncertainty into the evaluation of aerosol-cloud interactions. Second, following previous studies, we have used vertically uniform values in the prescribed CDNC scenarios designed to evaluate the aerosol-cloud interaction impact, because of the difficulty in measuring CDNC vertical distributions. To better assess the aerosol-cloud interaction impact on meteorology and air quality, it is important that the observation-based, vertically-resolved CDNC or CCN distributions be used in the construction of hypothetical pristine scenarios. Finally, while this study has quantified the impact of total anthropogenic aerosols on meteorology and air quality, the effects of aerosols from various sources could differ significantly due to distinct chemical compositions, which is a subject requiring further in-depth studies. In particular, a source-oriented version of WRF-Chem has been developed^45^ and coupled with the warm-cloud processes^46^, which represents a promising tool for the investigation of source-specific aerosol effects.

## Methods

### Model configurations

The modeling domain covers most of China except for some sparsely populated regions in westernmost and northernmost China (Supplementary Figure 1) with a horizontal grid resolution is 36 km × 36 km. The vertical resolution includes 24 layers from the surface to 50 mb with denser layers within the PBL. Major physical options used include the Grell-Freitas cumulus scheme^47^, the National Center for Environmental Prediction, Oregon State University, Air Force, and Hydrologic Research Lab’s (NOAH) land-surface module^48^, the Yonsei University (YSU) PBL scheme^49^, the Morrison double-moment scheme for cloud microphysics^50^, and the Fu-Liou-Gu (FLG) radiative transfer scheme^41, 51–53^. We apply a modified surface drag parameterization in the YSU PBL scheme^35^, which helps to reduce the positive wind speed bias.

In the model, we employ the Carbon-Bond Mechanism version Z (CBM-Z) gas-phase chemistry mechanism^54^. Rates for photolytic reactions are calculated using the Fast-J photolysis rate scheme^55^. The aerosol module used is the Model for Simulating Aerosol Interactions and Chemistry (MOSAIC)^56^, which includes all major aerosol processes except for the formation of secondary organic aerosols (SOA). SOA concentrations have been recognized to be significantly underestimated in most widely used chemical transport models^57, 58^. It is an important task to develop a comprehensive SOA module which well predicts SOA concentrations and their interactions with clouds, which is, however, beyond the scope of this study. The aqueous-phase chemistry is based on the Carnegie Mellon University (CMU) mechanism^59^.

To account for the aerosol direct effect, the aerosol optical properties including the layer optical depth, single scattering albedo, and asymmetry factor are calculated as a function of wavelength and three dimensional location, and then transferred to the FLG radiation scheme. The Lorenz-Mie theory is used to estimate the optical properties by assuming a core-shell mixing state^60, 61^. The first and second aerosol indirect effects are simulated following Tseng^62^. Specifically, aerosols are activated based on the parameterization of Abdul-Razzak and Ghan^63^, which is subsequently coupled with the Morrison two-moment cloud microphysics scheme. The prognostic cloud water content and effective radius calculated by the Morrison scheme are input into the FLG scheme for radiative transfer calculation. Note that prognostic aerosol concentrations are only considered for the indirect effect on grid-scale clouds. The interactions between aerosols and cumulus clouds have not been explicitly resolved. Also, INP distribution is only dependent on supersaturation in the current model^64^, and the effect of aerosols on ice nucleation is not explicitly accounted for.

The simulation period is January and July, 2013, representing winter and summer, respectively. Note that the mechanisms underlying our main finding that increased aerosols could lead to an additional enhancement in PM_2.5_ concentrations through a positive feedback loop induced by aerosol-cloud interactions hold true regardless of the simulation period, as shown in January and July cases. For this reason, simulations for two different seasons are sufficient to support the key finding of this study. A longer simulation period (such as a full year), however, might enable a better quantification of the magnitude of aerosol-cloud interaction impact, which is a subject requiring further studies. The meteorological initial and boundary conditions are derived from the National Centers for Environmental Prediction’s Final Analysis reanalysis data at 1.0° × 1.0° and 6-h resolution (http://rda.ucar.edu/datasets/ds083.2/). FDDA is not utilized in this study so as to allow full aerosol-cloud-radiation interactions. The initial and boundary conditions for gas and aerosol species are kept constant as the model default profile. A 7-day spin-up period is used to reduce the influence of initial conditions on modeling results.

Anthropogenic emissions in China have been developed by Tsinghua University for 2010 and 2012^65–68^, and subsequently updated to 2013 considering changes of activity data and air pollution control technologies. A unit-based method is applied to estimate emissions from large point sources including coal-fired power plants, iron and steel plants, and cement plants^69, 70^. The emissions in other countries are obtained from the MIX emission inventory^71^ for 2010, which is the latest year available. Biogenic emissions are calculated online using the Model of Emissions of Gases and Aerosols from Nature (MEGAN)^72^. Dust emissions are calculated online following Shao *et al*.^73^.

### Observational datasets and model evaluation protocols

We evaluate the model performance using a series of surface meteorology, surface air quality, and satellite observational datasets, which are summarized in Table S1 and briefly described below. For surface meteorological variables, we use observations obtained from the National Climatic Data Center (NCDC, http://www.ncdc.noaa.gov/), where hourly or 3-hour observations of WS10, T2, and Q2 are available for 380 sites distributed within the modeling domain. We also adopt gridded monthly precipitation datasets from the Global Precipitation Climatology Center (GPCC, http://www.esrl.noaa.gov/psd/data/gridded/data.gpcc.html), which are derived from quality-controlled station data. For surface air quality, we obtain measurements of hourly concentrations of major pollutants (PM_10_, PM_2.5_, SO_2_, NO_2_, and O_3_) from the Ministry of Environmental Protection of China (MEP, http://datacenter.mep.gov.cn/). Continuous measurements are available at 496 sites located in 74 major cities in China, including capital cities of all provinces and prefecture-level cities in three metropolitan regions (North China Plain, Yangtze River Delta, and Pearl River Delta). The observational data of PM_2.5_ chemical components are quite sparse and not publicly available during the simulation periods. In this study, we use the chemical component observations obtained during a field campaign period (from July 22–31, 2013) at two sites located in the North China Plain (see Supplementary Figure 1). Additionally, we compare model simulations with a series of satellite-based observations, including SWD and downward longwave radiation at surface (LWD) from the Clouds and the Earth’s Radiant Energy System (CERES, http://ceres.larc.nasa.gov/), NO_2_ vertical column density (http://www.temis.nl/airpollution/no2.html) from the Ozone Monitoring Instrument (OMI), and AOD, LWP, and CF from MODIS (http://ladsweb.nascom.nasa.gov/data/search.html) onboard the Terra satellite. We also derive CDNC from MODIS data following the method of Bennartz^25^ for comparison purposes.

We apply slightly different performance statistical indices for different datasets to facilitate inter-study comparison with previous studies. For model-measurement comparison of surface meteorological variables, we use statistical indices including mean observation (Mean Obs), mean simulation (Mean Sim), mean bias (MB), gross error (GE), root mean square error (RMSE) and index of agreement (IOA). The definitions and formulations of these variables are provided in Emery *et al*.^30^. The indices used for comparison with surface air quality observations are Mean Obs, Mean Sim, normalized mean bias (NMB), normalized mean error (NME), mean fractional bias (MFB), and mean fractional error (MFE), as documented in Boylan and Russell^36^. As for satellite observations, we adopt indices of Mean Obs, Mean Sim, NMB, NME, RMSE, and correlation coefficient (R). The vertically-resolved model outputs of gas, aerosol, and cloud variables except for CDNC are vertically integrated from the surface up to the model top to achieve the corresponding column variables. For CDNC, it is processed within low level warm clouds (850–950 hPa) following Bennartz^25^. Only model outputs that are closest to the satellite local overpassing time (14:00 for OMI and 11:00 for MODIS/Terra, Beijing time) are used in comparison to avoid sampling errors due to diurnal cycles.

### Data availability statement

All data needed to evaluate the conclusions in the paper are present in the paper and/or the Supplementary Information. Additional data related to this paper can be requested from the authors.

## Electronic supplementary material

Supplementary Information
