# Supplementary material for: Enhanced PM2.5 pollution in China due to aerosol-cloud interactions
Source: Sci Rep. 2017 Jun 30;7:4453. doi: 10.1038/s41598-017-04096-8 (PMC5493654; doi:10.1038/s41598-017-04096-8)
Supplement: Supplementary file 1 — Supplementary Information [file 41598_2017_4096_MOESM1_ESM.pdf]

Supplementary Materials for the paper entitled:

Enhanced $PM_{2.5}$ pollution in China due to aerosol-cloud interactions

Bin Zhao[1,*], Kuo-Nan Liou[1], Yu Gu[1], Qinbin Li[1], Jonathan Jiang[2], Hui Su[2], Cenlin He[1], Hsien-Liang R. Tseng[1], Shuxiao Wang[3,4], Run Liu[1], Ling Qi[1], Wei-Liang Lee[5], and Jiming Hao[3,4]

[1]Joint Institute for Regional Earth System Science and Engineering and Department of Atmospheric and Oceanic Sciences, University of California, Los Angeles, CA 90095, USA

[2]Jet propulsion Laboratory, California Institute of Technology, Pasadena, California 91109, USA

[3]State Key Joint Laboratory of Environment Simulation and Pollution Control, School of Environment, Tsinghua University, Beijing 100084, China

[4]State Environmental Protection Key Laboratory of Sources and Control of Air Pollution Complex, Beijing 100084, China

[5]Research Center for Environmental Changes, Academia Sinica, Taipei, Taiwan

Corresponding to: Bin Zhao (zhaob1206@ucla.edu)

1. Model evaluation

The model predictions agree fairly well with surface meteorological observations. The MBs in January/July are 0.79/0.42 m s^{-1}, −1.28/−1.17 K, −0.87/−1.06 g kg^{-1}, 2.3/11.6 mm $month^{-1}$ for WS10, T2, Q2, and precipitation, respectively. Emery et al.[1] proposed benchmark values for satisfactory performance for WS10, T2, and Q2: MB within ± 0.5 m s^{-1}, GE ≤ 2.0 m s^{-1}, RMSE ≤ 2.0 m s^{-1} and IOA ≥ 0.6 for WS10, MB within ±0.5 °C, GE ≤ 2.0 °C, and IOA ≥ 0.8 for T2, and MB within ±1.0 g kg^{-1}, GE of ≤ 2.0 g kg−1, and IOA ≥ 0.6 for Q2. We note that these benchmark values are proposed based on the performance of a series of model simulations with four dimensional data assimilation (FDDA). Nevertheless, FDDA is not utilized here to allow full aerosol-cloud-radiation interactions. Therefore, the model performance is not expected to be as good as those with FDDA. Table 2 in the main text shows that the performance statistics for WS10 in July and Q2 in January fall within benchmark ranges, and those for Q2 in July are very close to the benchmark ranges. The WS10 in January and T2 in both months exceed the benchmark range but still have smaller or similar biases compared with most previous WRF-Chem applications without FDDA over East Asia[2-7], in which MBs range from 0.4 to 3.1 m s^{-1} (mostly 1.2 to 2.6 m s^{-1}) for WS10 and from −1.8 to 1.0 K (mostly −1.8 to −0.8 K) for T2, respectively. Therefore, the model performance is considered to be decent.

With regard to surface air quality, the model performance for $PM_{2.5}$ has been described in the main text. For gaseous pollutants, the model-measurement agreement is decent for NO_2 and daily maximum O_3 concentrations in both months, and SO_2 concentrations in January, with NMBs within ±20%. The model overestimates SO_2 concentrations in July, likely attributable to the uncertainty in emission inventory and insufficient treatment of SO_2 oxidation reactions on dust surface[8] and on fine aerosols with high relative humidity and NH_3 neutralization[9,10]. The

observational data of $PM_{2.5}$ chemical components are quite sparse and not publicly available during the simulation periods. In this study, we compare with chemical component observations obtained during a field campaign period (from July 22nd-31st, 2013) at two sites located in the North China Plain (see Supplementary Figure 1). The comparison results are shown in Supplementary Figure 3. Simulated $PM_{2.5}$ concentrations agree fairly well with observations; NMBs are within ±6% for both sites. As for chemical components, NO_3^- concentration is overestimated (NMB = 6% to 52%), while SO_4^{2-} concentration is underestimated (NMB = −37% to −63%). There is a good agreement for NH_4^+ (NMB within ±23%) and total SNA (sulphate-nitrate-ammonium, NMB within ±23%). The overestimation of NO_3^- and underestimation for SO_4^{2-} are consistent with previous studies over East Asia, probably attributed to the lack of some chemical formation pathways in the modeling system [8,10,11]. Simulated elemental carbon (EC) concentrations approximately double observed EC concentrations. EC concentrations are strongly affected by local emissions, while the spatial distribution of our emission inventory (36 km × 36 km) may not be able to capture local emission sources surrounding observational sites, leading to model-observation bias. The overestimation may also be attributable to the absence of EC aging in WRF-Chem, which leads to reduced fraction of hydrophilic EC and thus reduced wet depsition. Finally, concentrations of organic carbon (OC) can be either underestimated or overestimated in these two sites. The current model does not include secondary organic aerosol (SOA) formation; inclusion will probably leads to higher simulated OC concentrations.

For the evaluation of cloud properties, the simulated cloud fraction (CF) agrees fairly well with observations over the ocean and in southern China, but significant underestimates occur in northern China, especially in January. The domain average NMBs are −32% and −9% in January

and July, respectively. The liquid water path (LWP) is substantially underestimated across the domain, with NMBs of −59% and −74% in January and July, respectively, which is a common problem for many previous chemical transport model simulations[2,5,12,13]. It is noted, however, the LWP retrieved from MODIS may be biased by a factor of 2 due to uncertainties in cloud particle size assumption[14]. The domain average cloud droplet number concentration (CDNC) is moderately underpredicted by 29% and 38% in January and July, respectively, with the most remarkable underestimates occurring on land in January. The discrepancies in cloud parameters may be related to several factors including aerosol number concentrations, water vapor, aerosol activation parameterization, cloud microphysics, and cumulus cloud schemes. For example, Zhang et al.[15] showed that the Abdul-Razzak and Ghan[16] aerosol activation parameterization used in this work tends to underpredict aerosol activation fraction and consequently underpredict CDNC and LWP compared with the Fountoukis and Nenes[17] parameterization. In addition, the significant underestimates of CF and LWP in northern China may be explained in part by the underestimates in water vapor mixing ratio. Finally, we note that large uncertainty in satellite-retrieved LWPs[14,18] and in CDNC estimation[19] may also partly account for the model-measurement discrepancy. Downward shortwave radiation at surface (SWD) and downward longwave radiation at surface (LWD) simulated by WRF-Chem agree fairly well with the CERES data in terms of both magnitude and spatial distributions, with NMBs of about 11% to 18% and −8% to −3%, respectively. The slight overestimates in SWD and underestimates in LWD are likely induced by the underestimates in LWP and AOD.

References

1. Emery, C., Tai, E. & Yarwood, G. *Enhanced meteorological modeling and performance evaluation for two texas episodes. Report to the Texas Natural Resources Conservation Commission*, <http://www.tceq.state.tx.us/assets/public/implementation/air/am/contracts/reports/mm/EnhancedMetModelingAndPerformanceEvaluation.pdf> (2001).
2. Cai, C. *et al.* Incorporation of new particle formation and early growth treatments into WRF/Chem: Model improvement, evaluation, and impacts of anthropogenic aerosols over East Asia. *Atmos. Environ.* **124**, 262-284, 10.1016/j.atmosenv.2015.05.046 (2016).
3. Jiang, F. *et al.* Regional modeling of secondary organic aerosol over China using WRF/Chem. *J. Aerosol. Sci.* **43**, 57-73, 10.1016/j.jaerosci.2011.09.003 (2012).
4. Zhang, B., Wang, Y. & Hao, J. Simulating aerosol-radiation-cloud feedbacks on meteorology and air quality over eastern China under severe haze conditions in winter. *Atmos. Chem. Phys.* **15**, 2387-2404, 10.5194/acp-15-2387-2015 (2015).
5. Chen, Y. *et al.* Application of an Online-Coupled Regional Climate Model, WRF-CAM5, over East Asia for Examination of Ice Nucleation Schemes: Part I. Comprehensive Model Evaluation and Trend Analysis for 2006 and 2011. *Climate* **3**, 627-667, 10.3390/cli3030627 (2015).
6. Zhang, Y. *et al.* Application of WRF/Chem over East Asia: Part I. Model evaluation and intercomparison with MM5/CMAQ. *Atmos. Environ.* **124**, 285-300, 10.1016/j.atmosenv.2015.07.022 (2016).
7. Zhang, Y. *et al.* Application of WRF/Chem over East Asia: Part II. Model improvement and sensitivity simulations. *Atmos. Environ.* **124**, 301-320, 10.1016/j.atmosenv.2015.07.023 (2016).
8. Fu, X. *et al.* Modeling analysis of secondary inorganic aerosols over China: pollution characteristics, and meteorological and dust impacts. *Sci. Rep-Uk.* **6**, 10.1038/srep35992 (2016).
9. Wang, G. H. *et al.* Persistent sulfate formation from London Fog to Chinese haze. *P. Natl. Acad. Sci. USA.* **113**, 13630-13635, 10.1073/pnas.1616540113 (2016).
10. Cheng, Y. F. *et al.* Reactive nitrogen chemistry in aerosol water as a source of sulfate during haze events in China. *Sci. Adv.* **2**, e1601530, DOI: 10.1126/sciadv.1601530 (2016).
11. Wang, Y., Zhang, Q. Q., He, K., Zhang, Q. & Chai, L. Sulfate-nitrate-ammonium aerosols over China: response to 2000-2015 emission changes of sulfur dioxide, nitrogen oxides, and ammonia. *Atmos. Chem. Phys.* **13**, 2635-2652, DOI 10.5194/acp-13-2635-2013 (2013).
12. Liu, X. Y., Zhang, Y., Zhang, Q. & He, M. B. Application of online-coupled WRF/Chem-MADRID in East Asia: Model evaluation and climatic effects of anthropogenic aerosols. *Atmos. Environ.* **124**, 321-336, 10.1016/j.atmosenv.2015.03.052 (2016).
13. Wang, K., Zhang, Y., Yahya, K., Wu, S.-Y. & Grell, G. Implementation and initial application of new chemistry-aerosol options in WRF/Chem for simulating secondary organic aerosols and aerosol indirect effects for regional air quality. *Atmos. Environ.* **115**, 716-732, 10.1016/j.atmosenv.2014.12.007 (2015).

14 Jiang, J. H. *et al.* Evaluation of cloud and water vapor simulations in CMIP5 climate models using NASA "A-Train" satellite observations. *J. Geophys. Res-Atmos.* **117**, 10.1029/2011jd017237 (2012).

15 Zhang, Y. *et al.* Development and initial application of the global-through-urban weather research and forecasting model with chemistry (GU-WRF/Chem). *J. Geophys. Res-Atmos.* **117**, 10.1029/2012jd017966 (2012).

16 Abdul-Razzak, H. & Ghan, S. J. A parameterization of aerosol activation. 3. Sectional representation. *J. Geophys. Res.* **107**, AAC1-1-6 (2002).

17 Fountoukis, C. & Nenes, A. Continued development of a cloud droplet formation parameterization for global climate models. *J. Geophys. Res-Atmos.* **110**, 10.1029/2004jd005591 (2005).

18 Seethala, C. & Horvath, A. Global assessment of AMSR-E and MODIS cloud liquid water path retrievals in warm oceanic clouds. *J. Geophys. Res-Atmos.* **115**, 10.1029/2009jd012662 (2010).

19 Bennartz, R. Global assessment of marine boundary layer cloud droplet number concentration from satellite. *J. Geophys. Res-Atmos.* **112**, D02201, 10.1029/2006jd007547 (2007).

Figures and Tables

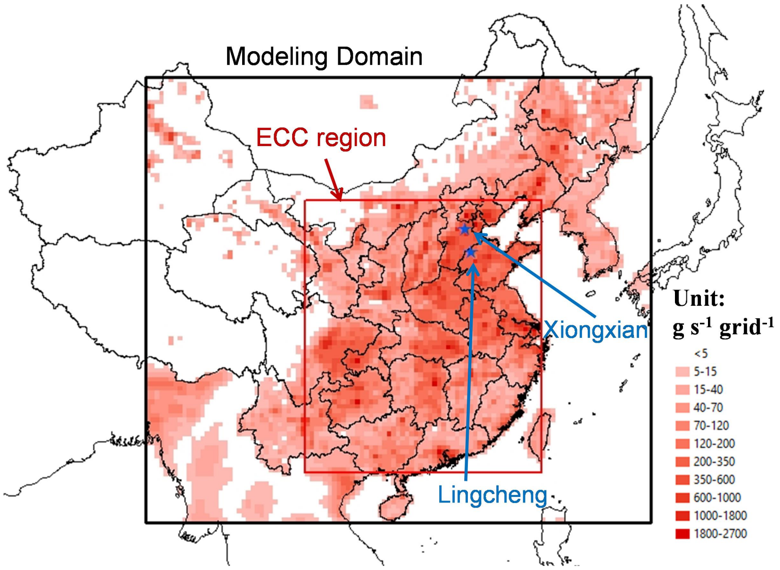

Supplementary Figure 1. The WRF-Chem modeling domain at a horizontal spatial resolution of 36 km (111 × 98 cells). The two blue stars indicate locations of the Xiongxian Site and the Lingcheng Site, where observations of $PM_{2.5}$ chemical components are available. The red rectangle indicates the Eastern and Central China (ECC). This region has high population density and high aerosol emissions and concentrations, and is thus the focus of this study. The colors represent primary $PM_{2.5}$ emission rates at 8:00 a.m. January 1st, 2013. This figure is produced using DotSpatial, [version 1.7], (http://dotspatial.codeplex.com/) and Microsoft PowerPoint 2013 (https://www.microsoft.com/).

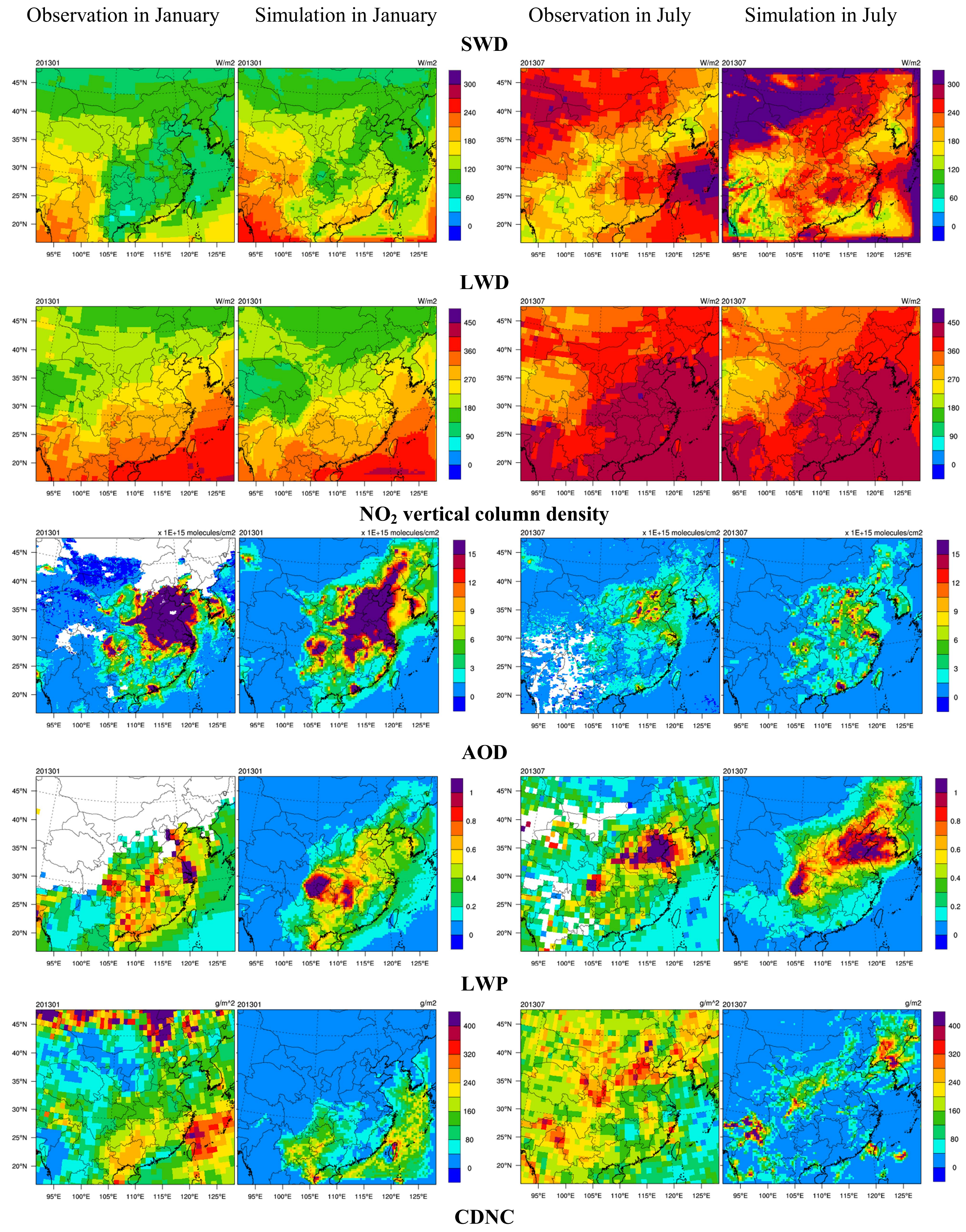

Observation in January
Simulation in January
Observation in July
Simulation in July
SWD
201301
W/m2
201307
LWD
NO2 vertical column density
x 1E+15 molecules/cm2
AOD
LWP
g/m^2
g/m2
CDNC

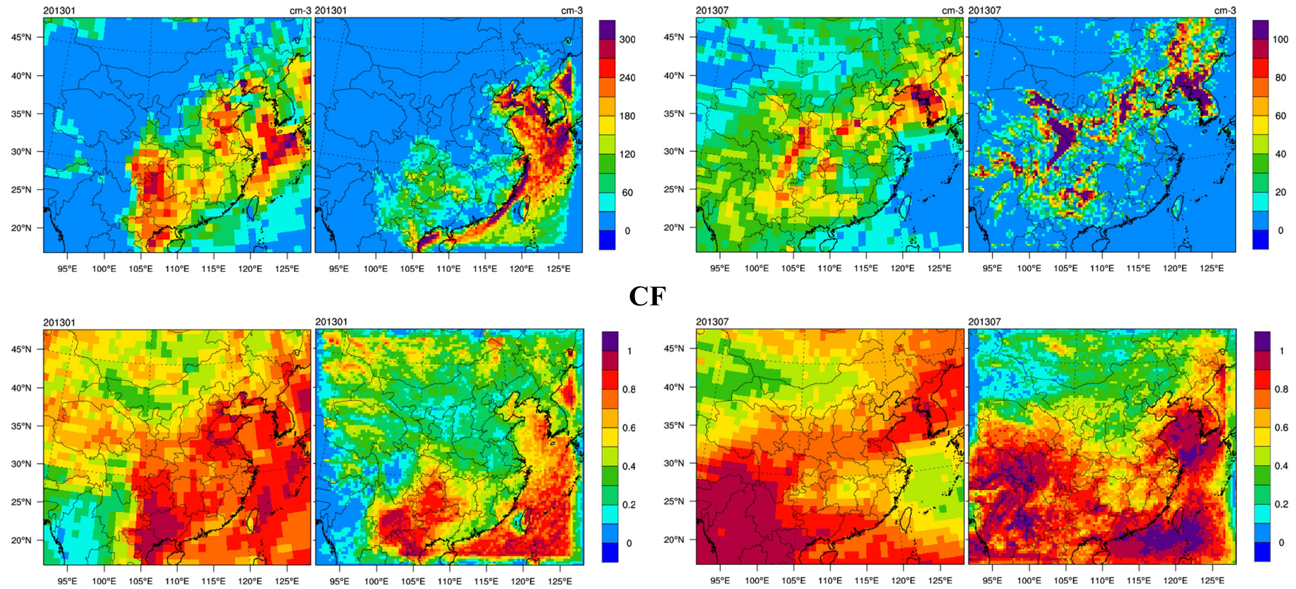

Supplementary Figure 2. Comparison of simulated SWD, LWD, NO_2 column, AOD, and cloud properties with satellite observations. This figure is produced using the NCAR Command Language (Version 6.2.1) [Software]. (2014). Boulder, Colorado: UCAR/NCAR/CISL/TDD. http://dx.doi.org/10.5065/D6WD3XH5.

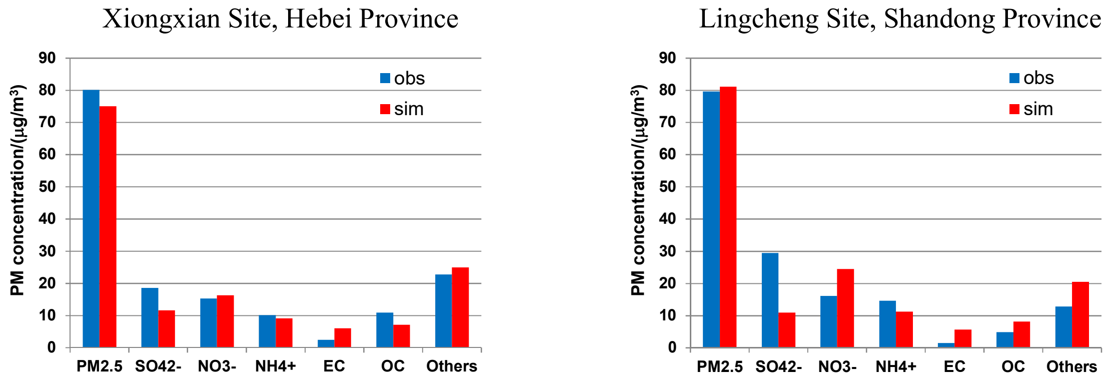

Supplementary Figure 3. Comparison of simulated concentrations of $PM_{2.5}$ and its chemical components with observations at the Xiongxian Site and the Lingcheng Site. This figure shows the average concentrations during July 22nd - July 31st.

Supplementary Table 1. Observational datasets used in model evaluation.

Type	Database	Variables	Sites/resolution	Frequency
Surface meteorology	NCDC	wind speed at 10 m (WS10), temperature at 2 m (T2), and water vapor mixing ratio at 2 m (Q2)	380 sites	Hourly or every 3 hour
	GPCC	precipitation	0.5º×0.5º	Monthly
Surface air quality	MEP	PM_{10}, $PM_{2.5}$, SO_2, NO_2, and O_3	496 sites in 74 large cities in China	Hourly
	$PM_{2.5}$ chemical components	$PM_{2.5}$, SO_4^{2-}, NO_3^-, NH_4^+, BC, and OC	2 sites: Xiongxian Site, Lingcheng Site	Daily
Satellite	CERES	Downward shortwave radiation at surface (SWD), downward longwave radiation at surface (LWD)	1º×1º	Monthly
	OMI	NO_2 vertical column density	0.125º×0.125º	Monthly
	MODIS/TERRA	aerosol optical depth (AOD), liquid water path (LWP), cloud fraction (CF), and cloud droplet number concentration (CDNC, derived from MODIS data)	1º×1º	Monthly
